# Wave reflections and global arterial compliance during normal human pregnancy

**DOI:** 10.14814/phy2.13947

**Published:** 2018-12-21

**Authors:** Claudia Rodriguez, Yueh‐Yun Chi, Kuei‐Hsun Chiu, Xiaoman Zhai, Melissa Lingis, Robert Stan Williams, Alice Rhoton‐Vlasak, Wilmer W. Nichols, John W. Petersen, Mark S. Segal, Kirk P. Conrad, Rajesh Mohandas

**Affiliations:** ^1^ Department of Animal Sciences University of Florida Gainesville Florida; ^2^ Department of Biostatistics University of Florida Gainesville Florida; ^3^ Division of Nephrology, Hypertension and Renal Transplantation University of Florida Gainesville Florida; ^4^ Department of Obstetrics and Gynecology University of Florida Gainesville Florida; ^5^ Division of Cardiovascular Medicine Department of Medicine University of Florida Gainesville Florida; ^6^ Nephrology and Hypertension Section Medical Service North Florida/South Georgia Veterans Health System Gainesville Florida; ^7^ Department of Physiology and Functional Genomics University of Florida Gainesville Florida

**Keywords:** Applanation tonometry, maternal cardiovascular function, pulse wave analysis, pulse wave velocity, SphygmoCor

## Abstract

Profound changes occur in the maternal circulation during pregnancy. Routine measures of arterial function – central systolic pressure (CSP) and augmentation index (AIx) – decline during normal human pregnancy. The objectives of this study were twofold: (1) explore wave reflection indices besides CSP and AIx that are not routinely reported, if at all, during normal human pregnancy; and (2) compare wave reflection indices and global arterial compliance (gAC) obtained from carotid artery pressure waveforms (CAPW) as a surrogate for aortic pressure waveforms (AOPW) versus AOPW synthesized from radial artery pressure waveforms (RAPW) using a generalized transfer function. To our knowledge, a comparison of these two methods has not been previously evaluated in the context of pregnancy. Ten healthy women with normal singleton pregnancies were studied using applanation tonometry (SphygmoCor) at pre‐conception, and then during 10–12 and 33–35 gestational weeks. CSP and AIx declined, and gAC increased during pregnancy as previously reported. As a consequence of the rise in gAC, the return of reflected waves of lesser magnitude from peripheral reflection sites to the aorta was delayed that, in turn, reduced systolic duration of reflected waves, augmentation index, central systolic pressure, LV wasted energy due to reflected waves, and increased brachial‐central pulse pressure. For several wave reflection indices, those derived from CAPW as a surrogate for AOPW versus RAPW using a generalized transfer function registered greater gestational increases of arterial compliance. This discordance may reflect imprecision of the generalized transfer function for some waveform parameters, though potential divergence of carotid artery and aortic pressure waveforms during pregnancy cannot be excluded.

## Introduction

Pregnancy is associated with marked changes in the maternal cardiovascular system especially during the first trimester. Several studies serially examined changes in central aortic blood pressures and augmentation index during human pregnancy beginning with a pre‐conception control (Table 1) (Robb et al. 2009; Fujime et al. 2012; Mahendru et al. 2014; Foo et al. 2017; Iacobaeus et al. 2017). Organs such as the brain, kidney, and heart are exposed to central aortic blood pressures, the magnitude of which is a strong predictor of adverse cardiovascular outcomes in nonpregnant individuals with and without hypertension (Vlachopoulos et al. 2010; Kollias et al. 2016; Cheng et al. 2017; Ochoa et al. 2018). In addition to central aortic blood pressures and augmentation index, analysis of the aortic pressure waveform provides a wealth of other information about reflected waves, wave reflection durations, cardiac efficiency, and global arterial compliance. To our knowledge, the majority of these variables available from aortic pressure waveform analysis has not been previously reported in pregnancy.

**Table 1:** Changes in wave reflection indices during normal pregnancy

References	No. subjects	Method	Control	Maternal Age (year)	Gestational age (week)	AIx@75 (%Δ)	Central SBP (%Δ)	Central PP (%Δ)
Robb et al. (2009)	22	Applanation tonometry using micromanometer (Milar instruments) & SphygmoCor (AtCor)	Postpartum (7 weeks)	30	16 24 32 37	Decreasing to nadir at 24 weeks compared to postpartum (*P* = 0.0002), then rising toward term (*P* < 0.01)	Decreasing to a nadir at 24 weeks compared to postpartum (*P* = n/a) rising thereafter (*P* = 0.13).	Decreasing to a nadir at 24 weeks compared to postpartum (*P* = n/a) rising thereafter (*P* < 0.001).
Fujime et al. (2012)	69	Automated applanation tonometry using HEM‐9000AI (Omron)	Postpartum (few days and 4 weeks)	31	12–14 23–27 34–36	Decreasing to a nadir at 23–27 week compared to postpartum (*P* < 0.05) rising thereafter (*P* < 0.05)	Decreasing to a nadir at 23–27 week compared to postpartum (*P* < 0.05) rising thereafter (*P* < 0.05)	n/a
Mahendru et al. (2014)	54	Applanation tonometry using SPC‐301 (Millar instruments) & SphygmoCor (AtCor)	Pre‐pregnant and Postpartum (14–17 weeks)	31	6–7 23–24 32–34	Decreasing to nadir at 23–24 weeks compared to Prepregnancy (*P* < 0.001), then slowly rising toward term (*P* < 0.001)	Decreasing at 6–7 weeks compared to Prepregnancy (*P* < 0.001), then slowly rising toward term (*P* < 0.001)	n/a
Foo et al. (2017)	140	Cuff‐based device Vicorder (Smart Medical)	Pre‐pregnant	33	6	Decreases by 3.1% (*P* = NS)	n/a	n/a
Iacobaeus et al. (2017)	52	Applanation tonometry using micromanometer& SphygmoCor (AtCor)	Postpartum (9 months)	32	11–14 22–24 32–34	Decreasing to a nadir at 24 weeks compared to postpartum (*P* < 0.001) rising thereafter (*P* < 0.001).	Decreasing to a nadir at 24 weeks compared to postpartum (*P* = NS) rising thereafter (*P* < 0.001).	Decreasing to a nadir at 34 weeks compared to postpartum (*P* < 0.01) rising thereafter (*P* < 0.05).

n/a = data not available.

Using applanation tonometry, the carotid artery pressure waveform is used as a surrogate for the aortic pressure waveform (Kelly et al. 1989; Kelly and Fitchett 1992; Chen et al. 1996; Poppas et al. 1997; Nichols et al. 2011a). The aortic pressure waveform is also derived from the radial artery pressure waveform using a proprietary generalized transfer function (Nichols et al. 2011a, 2015; Butlin and Qasem 2017). A high‐quality pressure waveform is typically easier to obtain from the radial than carotid artery. Thus, a generalized transfer function provides a convenient and reproducible aortic pressure waveform. However, the derivation and validation of a generalized transfer function necessitated concurrent, catheter‐based measurement of aortic pressure. Consequently, these studies were largely performed in an older population with stiffer vasculature, in whom catheterization was medically indicated (Nichols et al. 2011a; Butlin and Qasem 2017). Whether a generalized transfer function is applicable to young pregnant women with compliant vasculature has not been evaluated to the best of our knowledge.

One of the most characteristic vascular adaptations during pregnancy is an early and profound increase in arterial compliance (Poppas et al. 1997). Pulse wave analysis allows us to assess global arterial compliance, a measure of the pulsatile arterial load that is derived from the diastolic decay of the aortic pressure waveform and the systemic vascular resistance (Poppas et al. 1997). Noninvasive methods to measure arterial compliance could ultimately prove clinically useful for prediction of hypertensive complications of pregnancy (Khalil et al. 2014).

The objectives of this study were twofold. First, to perform a comprehensive study of pulse wave analysis including central aortic blood pressures, augmentation pressure due to reflected waves, wave reflection durations, as well as indices of cardiac efficiency and global arterial compliance during normal pregnancy beginning with a pre‐conception control. Second, to compare wave reflection indices obtained from the carotid artery pressure waveform as a surrogate for the aortic pressure waveform with those obtained from the aortic pressure waveform derived from the radial artery waveform using a generalized transfer function.

## Materials and Methods

### Study population

After written informed consent, subjects were recruited to participate in this study approved by the Institutional Review Board at the University of Florida. Ten healthy women with uncomplicated singleton pregnancies volunteered to join this study. The study participants were first evaluated pre‐conception during the follicular phase, 9.6 ± 0.5 days (mean ± SE) after the first day of their last menstrual period, and then during pregnancy at 12.0 ± 0.3 and 34.2 ± 0.3 weeks of gestation.

### Echocardiography

Echocardiograms were obtained in the left lateral decubitus position with an iE33 (Philips, The Netherlands) equipped with a broadband S5‐1 transducer (frequency transmitted 1.7 MHz, received 3.4 MHz) as previously described (Petersen et al. 2017). Doppler was performed from an apical five‐chamber orientation. Pulsed‐wave Doppler with placement of sample volume in the left ventricular (LV) outflow tract immediately proximal to the aortic valve cusps was used to determine LV outflow tract VTI (velocity time integral). LV outflow VTI was measured in up to five different beats. LV outflow tract cross‐sectional area was calculated after measuring the diameter of the outflow tract on parasternal images. LV outflow cross‐sectional area was determined up to five different beats. VTI data were used to estimate stroke volume and CO. The VTI of each beat was multiplied by the estimated average LV outflow cross‐sectional area (determined by averaging the measured diameters of all beats). The average CO for up to five beats was then determined (Petersen et al. 2017).

### Pulse wave analysis

Wave reflection characteristics and event timing were assessed noninvasively from carotid and central aortic pressure waveforms, the latter synthesized from radial artery waveforms using a generalized transfer function (SphygmoCor CvMS; AtCor) (Nichols et al. 2011a; Butlin and Qasem 2017). After at least 20 min of supine rest and within 5 min of peripheral blood pressure and heart rate determination (oscillometric sphygmomanometer; Datascope), a high‐fidelity micromanometer (Millar Instruments, Houston, Tx) was placed on the radial artery to capture valid, reproducible waveforms based on the software's quality control parameters (average pulse height ≥80, pulse height variation ≤5%, diastolic variation ≤5%, shape variation ≤4%, and overall Operator Index ≥80%). Multiple, consecutive peripheral (radial) pressure waveforms were averaged and processed via a generalized transfer function to derive the central aortic pressure waveform and subsequent indices of cardiovascular function (Nichols et al. 2011a). For comparison, these values were also derived from multiple, consecutive waveforms taken with the micromanometer at the level of the carotid artery; however, in this case a generalized transfer function was not utilized. Pressure waveform calibration was performed as follows: for the radial artery assessments, systolic and diastolic pressure readings from Datascope were entered into the SphygmoCor software package just prior to capture of radial artery pressure waveforms; for the carotid artery assessments, diastolic pressure reading from Datascope and mean arterial pressure taken from the derived central aortic pressure waveform (from generalized transfer function processing of the radial artery pressure waveforms) were entered into the SphygmoCor software package just prior to capture of carotid artery pressure waveforms. Assessments that did not meet all of SphygmoCor software's intended quality control criteria were excluded during a blinded, offline vetting process.

**Figure 1:**
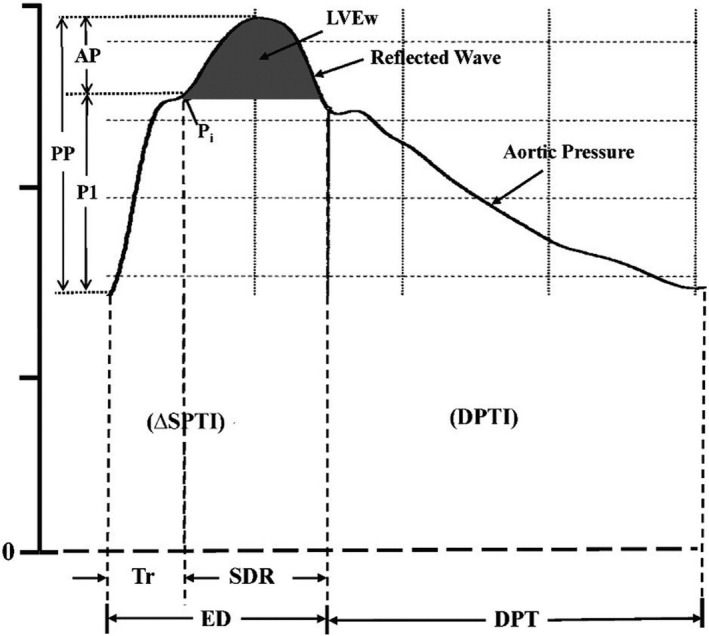
Central aortic pressure waveform synthesized from a radial pressure waveform. *P*_i_ indicates the merging (or inflection) point of the forward traveling and reflected (or backward traveling) waves. The early part of the ascending aortic pressure (i.e., forward traveling) wave with amplitude (P1) is generated by left ventricular (LV) ejection. The later part of the pressure wave with amplitude (AP) is the reflected wave arriving during systole and adding to the forward traveling pressure wave. Thus, pulse pressure (PP) = P1 + AP and augmentation index (AIx) = AP/PP. Tr is the sum of the travel time of the forward traveling wave from the LV to the periphery and the backward traveling reflected wave from the periphery to the LV; SDR is systolic duration of the reflected wave; ED is ejection duration (or systolic pressure time, SPT); DPTI is diastolic pressure time integral (or index) and DPT is diastolic pressure time. The area under the systolic portion of the reflected wave (dark shaded area) is defined as LV wasted energy (LVEw). Systolic pressure time index (SPTI) = ΔSPTI + LVEw. From Nichols et al. (2015) with permission.

Figure 1 depicts a schematic of the aortic pressure waveform and defines the various indices of wave reflections we measured in this study (Nichols et al. 2015).

### Global arterial compliance

**Figure 2:**
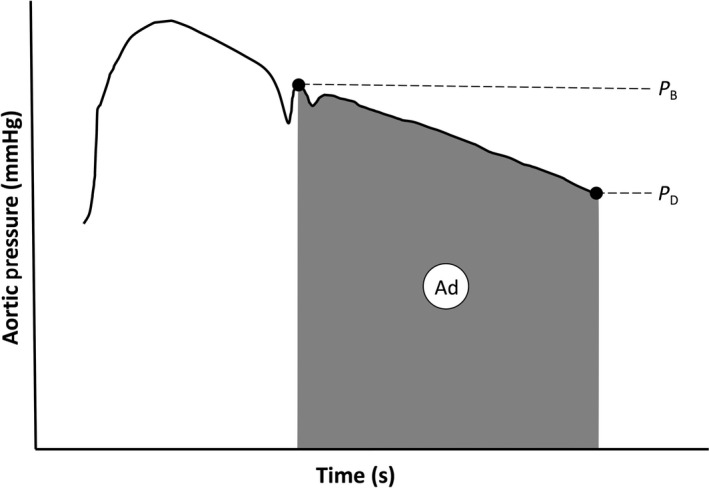
Estimation of global arterial compliance using the area method (AC_area_). A central aortic pressure waveform obtained from the carotid artery pressure waveform as a surrogate or synthesized from the radial artery pressure waveform using a generalized transfer function is depicted. PB and PD define the beginning and end of the diastolic portion of the aortic pressure waveform. Ad is the area under the curve defined by these boundaries. AC_area_ = Ad/[SVR(PB‐PD)], where SVR is systemic vascular resistance calculated by MAP/CO, and CO obtained by echocardiography (see Materials and Methods for further details). Based on Liu et al. (1986) and Poppas et al. (1997).

Two methods to measure global arterial compliance were used. First, global arterial compliance (AC_area_) was derived from the diastolic decay of the aortic pressure waveform and the systemic vascular resistance as shown in Figure 2 (Liu et al. 1986; Poppas et al. 1997; Conrad et al. 2004). AC_area_ = Ad/[SVR(*P*_B_−*P*_D_)], where *P*_B_ and *P*_D_ are the pressures at the beginning and end of the diastolic decay curve, respectively, and Ad is the area under the curve over this region. Note, however, that SphygmoCor reports *P*_ESP_ (end systolic pressure), but not *P*_B_. Therefore, we substituted *P*_B_ with *P*_ESP_, which slightly underestimates the beginning of the diastolic decay curve, being at the nadir of the dicrotic notch rather than at the true start of the diastolic decay curve. SVR was calculated as MAP/CO. The second method of global arterial compliance was calculated as stroke volume/central pulse pressure (Chemla et al. 1998). Stroke volume was calculated by the VTI × aortic outflow tract area.

### Pulse wave velocity

Applanation tonometry (SphygmoCor CvMS; AtCor) was again utilized to assess carotid, femoral, and radial pressure waveforms noninvasively; and pulse wave velocity (PWV) was calculated using standard methods after at least 10 min of supine rest and within 5 min of peripheral blood pressure and heart rate determination (Datascope). Prior to the assessments, the travel distance was measured between recording sites using a nonstretchable medical tape measure and a caliper. The carotid‐radial distance was measured from the suprasternal notch to the site of radial artery waveform measurement at the wrist. The carotid‐femoral distance was measured from the suprasternal notch to the site of femoral artery waveform measurement using a caliper to avoid effects of body size and/or shape (Levi‐Marpillat et al. 2013). The distance between the suprasternal notch and the site of waveform measurement on the right carotid artery was also measured with the tape. All distances were entered into the SphygmoCor software for determination of vascular path length (distal minus proximal distances, D) for carotid‐radial (cr) and carotid‐femoral (cf), respectively. The carotid and femoral or radial pressure waveforms were aligned by a concurrently measured ECG over a capture period of 10 sec, and the transit times were calculated from the foot‐to‐foot time difference between the carotid and femoral or carotid and radial pressure waveforms (PWTT). PWTT and D were exported by the software. At least two consecutive measurements were taken for each pair (carotid‐radial or carotid‐femoral) of arterial sites (Weber et al. 2009).

Because each subject was typically evaluated on several occasions over a period of months, multiple distance measurements were available for each subject. These distances were averaged across the visits. This average distance was then used to compute PWV at each of the visits for each subject (D/PWTT).

PWV assessments that did not meet all of SphygmoCor software's intended quality control criteria were excluded during a blinded, offline vetting process. AtCor defines assessments that meet Quality Control criteria as: (1) standard deviation across waveforms within each assessment is 6% or less of the Mean Time for both proximal (carotid) and distal (radial or femoral), (2) heart rates determined at each site within a given assessment are within 5 beats per minute of each other, and (3) standard deviation of the transit time is 10% or less.

### Statistics

Numerical data are presented as mean ± SE (Tables) or mean ± 1.96SE (Figures). Linear mixed models were used to relate features derived from the pulse wave analyses with the method (Method: carotid vs. radial) and weeks in gestation (Time, treated as a categorical predictor). The model accounts for the correlations for measurements taken over time and allows for missing data. The full model included the two main effects and the interaction between Method and Time. The software SAS (9.4) and R (2.15) were used for the analyses.

## Results

### Subjects

The demographic and clinical characteristics of subjects studied have been previously reported (Petersen et al. 2017). Briefly, the study population had a mean age at the start of pregnancy of 32.5 ± 2.0 years and were all non‐Hispanic, Caucasian even though they were selected randomly from a larger population of women studied. All subjects were healthy with uncomplicated pregnancies. The mean prepregnancy weight and body mass index were 68 ± 6.5 kg and 25 ± 2.4 kg·m^−2^, respectively.

### Peripheral and central blood pressures

**Figure 3:**
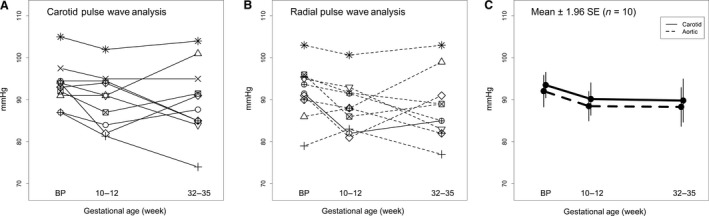
Central aortic systolic pressure for each subject (*N* = 10) using (A) carotid artery or (B) aortic pressure waveforms, the latter derived from radial artery pressure waveforms using a generalized transfer function. (C) Mean ± 1.96SE.

Systolic, diastolic, and mean peripheral and central blood pressures declined during pregnancy, but did not reach significance (Table 2 and 3; Fig. 3). However, the pulse pressure amplification ratio rose significantly (*P* = 0.007, Table 2). Central systolic blood pressures fell ~4 mmHg early in pregnancy and remained decreased through late gestation (Fig. 3), and central diastolic blood pressure also declined during the first trimester by 3–5 mmHg, but tended to rise in late pregnancy. Comparable central aortic blood pressures were observed whether obtained from the carotid artery waveform or from the aortic pressure waveform synthesized from the radial artery pressure waveform using a generalized transfer function (Table 3).

**Table 2:** Peripheral pressures

Variable	Pre‐Pregnant		10–12 weeks		33–35 weeks		*P*‐value
	Mean	SE	Mean	SE	Mean	SE	Time
Systolic (mmHg)	103	1	102	2	102	2	0.46
Diastolic (mmHg)	65	1	61	1	63	2	0.59
Mean (mmHg)	77	1	74	1	74	2	0.23
Pulse Pressure (mmHg)	38	1	41	1	38	2	0.90
PPAmpRatio^a^	145	5	156	4	159	3	0.007

a PPAmpRatio, brachial/central pulse pressures, or pulse pressure amplification.

**Table 3:** Central pressures

Variable	Pre‐pregnant				10–12 weeks				33–35 weeks				*P*‐value		
	Carotid		Aortic		Carotid		Aortic		Carotid		Aortic		Mixed model		
	Mean	SE	Mean	SE	Mean	SE	Mean	SE	Mean	SE	Mean	SE	Method	Time	Method*Time
Systolic (mmHg)	94	2	92	2	90	2	88	2	90	3	88	2	0.07	0.21	0.97
Diastolic (mmHg)	66	1	65	2	61	1	62	2	63	2	64	2	0.85	0.16	0.29
Mean (mmHg)	78	1	77	1	74	2	74	2	74	2	74	2	0.56	0.18	0.43
Pulse Pressure (mmHg)	27	2	27	1	29	1	27	1	27	2	24	2	0.02	0.35	0.13
End Systolic Pressure (mmHg)	82	1	83	2	76	2	78	2	75	2	78	3	0.01	0.02	0.68

Carotid, carotid artery waveform. Aortic, radial artery pressure waveform using a generalized transfer function to derive aortic pressure waveform.

### Augmentation pressures

**Figure 4:**
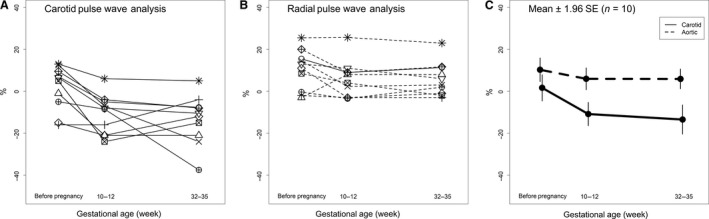
Augmentation index normalized to heart rate of 75 b/min for each subject (*N* = 10) using (A) carotid artery or (B) aortic pressure waveforms, the latter derived from radial artery pressure waveforms using a generalized transfer function. (C) Mean ± 1.96SE. Method: *P* = 0.003; Time: *P* = 0.004; Method × Time: *P* = 0.03.

Augmentation pressures (AP) and AP normalized to a heart rate of 75 beats per min decreased starting in early pregnancy reaching a nadir in late pregnancy (*P* = 0.001 and 0.002 vs. prepregnancy, respectively; Table 4). Augmentation index (AIx) and Alx normalized to 75 beats per min also dropped starting in early pregnancy (*P* = 0.001 and 0.004, respectively; Table 4, Fig. 4). Whether obtained from the carotid artery or aortic pressure waveform using a generalized transfer function, AP and AIx both showed gestational declines (vide supra). However, AP and AIx fell significantly more during pregnancy when ascertained from the carotid artery pressure waveform even reaching negative values (Type C waveform (Nichols et al. 2011b)) (Method × Time all *P* < 0.05; Table 4, Fig. 4). As described in Figure 1 (Nichols et al. 2011b), augmentation index was calculated from the pressure waveform. The augmentation index is negative in the type C waveform because the inflection point or beginning upstroke of the reflected wave occurs after peak systolic pressure (Nichols et al. 2011b).

**Table 4:** Augmentation pressures

Variable	Pre‐pregnant				10–12 weeks				33–35 weeks				*P*‐value		
	Carotid		Aortic		Carotid		Aortic		Carotid		Aortic		Mixed Model		
	Mean	SE	Mean	SE	Mean	SE	Mean	SE	Mean	SE	Mean	SE	Method	Time	Method*Time
Augmentation Pressure (mmHg)	2.3	1.0	4.5	1.0	−3.0	0.8	2.6	0.8	−4.0	1.1	2.0	0.6	0.001	0.001	0.03
Augmentation Pressure @HR75 (mmHg)	1	1	3	1	−3	1	2	1	−4	1	2	1	0.004	0.002	0.03
Augmentation Index (%)	7	4	16	3	−9	3	9	2	−14	4	6	2	0.001	0.001	0.02
Augmentation Index @HR75 (%)	1	3	10	3	−11	3	6	3	−13	4	6	3	0.003	0.004	0.03
P1 Height (mmHg)	24	1	22	0	29	2	24	1	27	2	23	1	0.001	0.049	0.09

P1 Height, Amplification of forward pressure wave.

### Wave reflection durations

**Figure 5:**
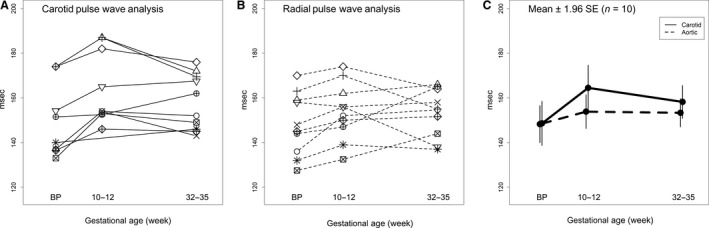
Round‐trip travel time of the pressure wave to and from major reflecting sites for each subject (*N* = 10) using (A) carotid artery or (B) aortic pressure waveforms, the latter derived from radial artery pressure waveforms using a generalized transfer function. (C) Mean ± 1.96SE. Time: *P* = 0.010; Method × Time: *P* = 0.10

**Figure 6:**
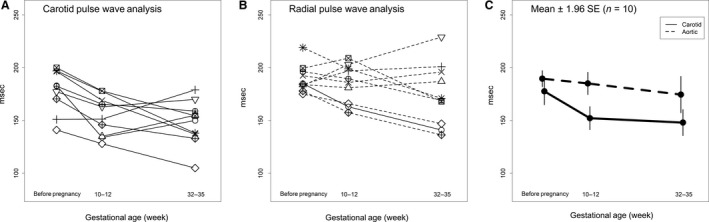
Systolic duration of the reflected wave for each subject (*N* = 10) using (A) carotid artery or (B) aortic pressure waveforms, the latter derived from radial artery pressure waveforms using a generalized transfer function. (C) Mean ± 1.96SE. Method: *P* = 0.001; Time: *P* = 0.012; Method × Time: *P* = 0.03.

Round‐trip travel time of the pressure wave to and from major reflection points (Tr) increased during pregnancy beginning in the first trimester (*P* = 0.01; Table 5, Fig. 5). This increase tended to be greater when determined by the carotid artery pressure waveform than aortic pressure waveform derived from the radial artery pressure waveform using a generalized transfer function (Method × Time *P* = 0.10). Systolic duration of reflected waves (SDR) decreased throughout pregnancy (*P* = 0.012); however, a more marked decline was observed for SDR derived from the carotid artery pressure waveform (Method × Time *P* = 0.03; Table 5, Fig. 6).

**Table 5:** Durations

Variable	Pre‐pregnant				10–12 weeks				33–35 weeks				*P*‐value		
	Carotid		Aortic		Carotid		Aortic		Carotid		Aortic		Mixed model		
	Mean	SE	Mean	SE	Mean	SE	Mean	SE	Mean	SE	Mean	SE	Method	Time	Method*Time
HR (b/min)	64	1	63	2	71	2	69	2	77	3	74	3	0.10	0.002	0.40
RR Duration (msec)	948	30	957	27	860	30	880	28	791	30	822	32	0.11	0.003	0.58
Ejection Duration (msec)	326	3	338	3	316	5	339	5	306	8	328	9	0.001	0.06	0.12
Ejection Duration Period (%)	35	1	35	1	37	1	39	1	39	1	40	1	0.01	0.003	0.58
Diastolic Duration Period (%)	65	1	65	1	63	1	61	1	61	1	60	1	0.01	0.002	0.62
SPTI (mmHg·sec)	1819	48	1817	47	1859	59	1904	54	1938	69	1976	76	0.12	0.17	0.19
DPTI (mmHg·sec)	2861	57	2807	82	2575	68	2527	62	2526	97	2494	94	0.15	0.006	0.91
Tr (msec)	149	5	148	4	165	5	154	4	159	4	153	3	0.17	0.01	0.10
SDR (msec)	178	6	190	4	152	6	185	5	148	6	175	9	0.001	0.01	0.03

SPTI, systolic pressure time index; DPTI, diastolic pressure time index; Tr, Round‐trip travel time of the pressure wave to and from major reflection site; SDR, systolic duration of reflected wave.

### Myocardial efficiency

**Figure 7:**
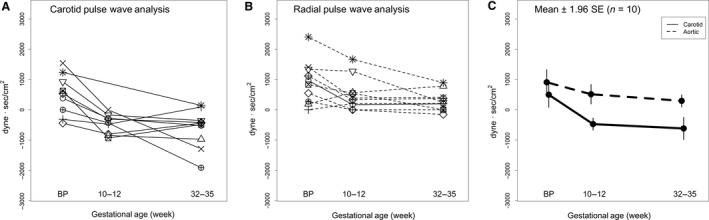
Left ventricular wasted energy for each subject (*N* = 10) using (A) carotid artery or (B) aortic pressure waveforms, the latter derived from radial artery pressure waveforms using a generalized transfer function. (C) Mean ± 1.96SE. Method: *P* = 0.01; Time: *P* = 0.001; Method × Time: *P* = 0.13.

Left ventricular energy wasted due to wave reflections declined throughout pregnancy (*P* = 0.001; Table 6, Fig. 7). Although the decline during pregnancy was similar between the two methods of measurement (Method × Time *P* = 0.13), across all time points, values derived from the carotid artery were lower (*P* = 0.001). Two indices of myocardial oxygen supply and demand, the subendocardial viability ratio (SEVR) and diastolic‐systolic pressure time fraction ratio (DPTF/SPTF), decreased during pregnancy (both *P* = 0.002; Table 6). The gestational changes in these two indices of myocardial oxygen supply and demand were comparable between the two methods of measurement (Method × Time *P* = 0.67 and 0.60, respectively), although overall, values derived from the carotid artery pressure waveform were higher (*P* = 0.08 and 0.02, respectively).

**Table 6:** Myocardial efficiency**.**

Variable	Pre‐Pregnant				10–12 weeks				33–35 weeks				*P*‐value		
	Carotid		Aortic		Carotid		Aortic		Carotid		Aortic		Mixed model		
	Mean	SE	Mean	SE	Mean	SE	Mean	SE	Mean	SE	Mean	SE	Method	Time	Method*Time
SVER	159	6	156	7	140	5	133	4	131	5	128	6	0.08	0.002	0.67
LV wasted energy (dyne·sec/cm^2^)	500	212	910	215	−472	102	515	164	−616	187	296	101	0.001	0.001	0.13
DPTF/SPTF	1.9	0.1	1.8	0.1	1.7	0.1	1.6	0.1	1.6	0.1	1.5	0.1	0.02	0.002	0.60

SVER, subendocardial viability ratio (DPTI/SPTI); DPTF/SPTF, systolic/diastolic pressure time fraction reflects myocardial oxygen supply/demand.

### Global artery compliance

**Figure 8:**
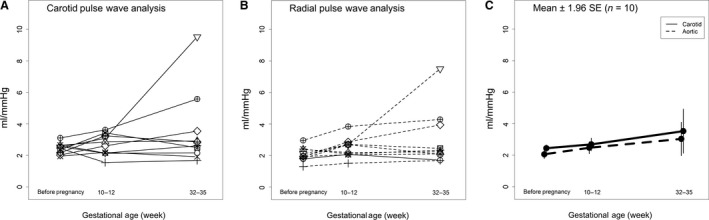
Global arterial compliance for each subject (*N* = 10) using (A) carotid artery or (B) aortic pressure waveforms, the latter derived from radial artery pressure waveforms using a generalized transfer function. (C) Mean ± 1.96SE. Method: *P* = 0.01; Time: *P* = 0.13.

Global arterial compliance obtained from the carotid artery pressure waveform as determined by the area method was overall ~0.5 mmHg/L higher relative to that obtained from the aortic pressure waveform synthesized from the radial artery pressure waveform using a generalized transfer function (*P* = 0.01; Table 7). The major independent variable driving this difference was ESP‐PD (*P* = 0.001; Table 7). However, both methods exhibited a comparable rise of ~1 mmHg/L (~40% increase) during pregnancy (Method × Time *P* = 0.41; Table 7, Fig. 8). Although the magnitude of increase of global arterial compliance during pregnancy was less when assessed by the ratio of stroke volume/central pulse pressure, in this case, the values obtained from the aortic pressure waveform using a generalized transfer function were modestly greater (Method × Time *P* = 0.05; Table 7).

**Table 7:** Global arterial compliance

Variable	Pre‐pregnant				10–12 weeks				33–35 weeks				*P*‐value		
	Carotid		Aortic		Carotid		Aortic		Carotid		Aortic		Mixed model		
	Mean	SE	Mean	SE	Mean	SE	Mean	SE	Mean	SE	Mean	SE	Method	Time	Method*Time
Global AC (mL/mmHg)	2.4	0.1	2.1	0.1	2.7	0.2	2.5	0.2	3.5	0.7	3.0	0.5	0.01	0.13	0.41
Ad (mmHg·min)	46	2	45	2	37	2	37	2	33	2	34	2	0.83	0.001	0.66
ESP‐DP (mmHg)	15	1	18	1	15	1	16	1	12	1	14	2	0.001	0.03	0.25
SVR (mmHg/mL*min^−1^)	1261	65	1261	65	985	69	985	69	999	70	999	70		0.005	
SV/PP (mL/mmHg)	2.7	0.1	2.7	0.1	2.7	0.1	2.9	0.2	3.1	0.3	3.4	0.3	0.03	0.09	0.05

Global AC, global arterial compliance; Ad, area under the diastolic decay of the aortic waveform (diastolic time integral/heart rate); ESP‐DP, end systolic–diastolic pressure; SVR, systolic vascular resistance. Note that Ad, ESP‐PD, and SVR are the independent variables used to calculate Global AC. SVR was derived from MAP/CO, CO obtained by echocardiography (see Methods). SV/PP, stroke volume/central aortic pulse pressure.

### Pulse wave velocity

Both carotid‐femoral and carotid‐radial pulse wave velocities decreased during pregnancy (*P* = 0.04 and 0.08, respectively; Table 8).

**Table 8:** Pulse wave velocity

Variable	Pre‐Pregnant				10–12 weeks				33–35 weeks				*P*‐value	
	Femoral		Radial		Femoral		Radial		Femoral		Radial		Femoral	Radial
	Mean	SE	Mean	SE	Mean	SE	Mean	SE	Mean	SE	Mean	SE	Time	Time
PWV (m/sec)	5.9	0.2	7.9	0.6	5.3	0.2	7.0	0.5	5.4	0.3	7.2	0.5	0.04	0.08

Femoral, carotid to femoral PWV; Radial, carotid to radial PWV.

## Discussion

Our results corroborated previous investigations demonstrating a fall in central blood pressures and augmentation indices during normal human pregnancy (Table 1) (Robb et al. 2009; Fujime et al. 2012; Mahendru et al. 2014; Foo et al. 2017; Iacobaeus et al. 2017). We further showed elevated round‐trip travel time from major reflection sites and reduced systolic duration of reflected waves resulting in decreased left ventricular wasted energy due to reflected waves. Consistent with these observations were increased pulse pressure amplification, and as previously reported (Poppas et al. 1997; Debrah et al. 2006; Oyama‐Kato et al. 2006; Iacobaeus et al. 2017), elevated global arterial compliance and reduced central and peripheral pulse wave velocities during pregnancy. Of further note, we observed significant differences in the magnitude of many, but not all wave reflection indices obtained from carotid artery pressure waveforms used as a surrogate for aortic pressures waveforms versus aortic pressure waveforms synthesized from radial artery pressure waveforms using a generalized transfer function. On balance, the results obtained from carotid artery pressure waveforms indicated a more compliant circulation during pregnancy.

To our knowledge, this is the first report to comprehensively examine longitudinal changes in wave reflection indices, durations, and myocardial efficiency during normal human pregnancy. Indeed, the gestational decrease in augmentation indices resulted from reflected waves of lesser amplitude returning later in the cardiac cycle after peak systole due to a more compliant circulation. The increase in round‐trip travel time of reflected waves to and from major reflecting sites and decrease in systolic duration of the reflected waves correspondingly reduced energy wasted by the heart due to the reflected waves. Although this improved efficiency of ventricular‐arterial coupling in pregnancy is salutary for the heart, measures reflecting myocardial oxygenation suggested a deterioration in the oxygen supply/demand ratio during gestation. This conclusion arose from reductions in both the diastolic–systolic pressure time index and diastolic–systolic time fraction ratios, and may be one factor that contributes to unmasking of occult ischemic heart disease during pregnancy precipitating myocardial infarction (Turitz and Friedman 2014; Smilowitz et al. 2018).

Using the pre‐pregnant state as control, we corroborated earlier work demonstrating an increase in global arterial compliance during normal human pregnancy, in which the postpartum state was the control (Poppas et al. 1997). Fewer number of subjects in our study (*n* = 10) likely precluded statistical significance (*P* = 0.13 and 0.094 for AC_area_ and SV/PP methods, respectively). Nevertheless, by using the same AC_area_ method as Poppas et al. (1997), we also showed ~40% increase in global arterial compliance. Further supporting the finding of increased arterial compliance during pregnancy was a reduction in carotid‐femoral and carotid‐radial pulse wave velocities as previously reported (Oyama‐Kato et al. 2006; Iacobaeus et al. 2017), although the nadir in pulse wave velocity was earlier in pregnancy than the peak in global arterial compliance.

Another goal of this work was to compare pulse wave analysis obtained from the directly measured carotid pressure waveforms with derived aortic pressure waveforms. For obvious, ethical reasons one cannot obtain direct, catheter‐based aortic pressures in normal pregnant women to compare with aortic pressures derived from radial artery pressure waveforms to validate applicability of a generalized transfer function to pregnancy. However, the carotid artery and aortic pressure waveforms were previously reported to be comparable (Kelly et al. 1989; Kelly and Fitchett 1992; Chen et al. 1996; Poppas et al. 1997; Nichols et al. 2011a). Although direction of change was consistent between the two methods, there was discordance in the magnitude of change during pregnancy for some, but not all indices of wave reflections. On one hand, there were significant Method x Time interactions for augmentation indices and systolic duration of reflected waves with differences trending for round‐trip travel time of reflected waves to and from reflection sites (*P* = 0.10) and left ventricular wasted energy (*P* = 0.13). On the other hand, gestational changes in central systolic pressure and global arterial compliance, the latter as assessed by the area method, were comparable between the two approaches, although overall central aortic systolic pressure trended to be lower (*P* = 0.07) and global arterial compliance was significantly higher (*P* = 0.01) as assessed by carotid artery waveforms. Taken together, the wave reflection indices derived from carotid pressure waveforms yielded a more compliant circulation in general and/or larger gestational increase of arterial compliance. Assuming the carotid pressure waveform to be a valid surrogate for the aortic pressure waveform in pregnancy as it is in the nonpregnant condition (vide supra), then our results suggested that there may be imprecision of the generalized transfer function for some wave reflection indices. However, we could not exclude the possibility that there may be some discordance between the carotid artery and aortic pressure waveforms during pregnancy. Conceivably, both explanations were at play. Our study provides a comprehensive and detailed characterization of pulse wave analysis during pregnancy, and establishes methodology for use in future studies of pregnancy. Detecting circulatory abnormalities including of pulse wave analysis in early gestation may ultimately be useful in the prediction and prevention of pregnancy disorders such as preeclampsia and fetal growth restriction.

## Conflict of Interest

The authors declare that no conflict of interest exists.
